# Sensitivity and specificity of routine diagnostic work-up for tuberculosis in lung clinics in Yogyakarta, Indonesia: a cohort study

**DOI:** 10.1186/s12889-019-6658-8

**Published:** 2019-04-02

**Authors:** Antonia Morita I. Saktiawati, Yanri W. Subronto, Ymkje Stienstra, Sumardi, Fabiola Supit, Tjip S. van der Werf

**Affiliations:** 1 Faculty of Medicine, Public Health and Nursing, Universitas Gadjah Mada, Jalan Farmako no.1, Sekip Utara, Yogyakarta, 55281 Indonesia; 2 University of Groningen, University Medical Center Groningen, Department of Internal Medicine/ Infectious Diseases, Groningen, The Netherlands; 3 Center for Tropical Medicine, Universitas Gadjah Mada, Yogyakarta, Indonesia

**Keywords:** Diagnosis, Tuberculosis, Sensitivity, Specificity, Culture, Microscopy, Radiography

## Abstract

**Background:**

Establishing a correct diagnosis is challenging. We aimed to investigate the sensitivity and specificity of routine tuberculosis (TB) diagnostic work-up in lung clinics in Indonesia, a country with the third highest TB burden and the second highest gap between notifications of TB cases and the best estimate of incident cases in the world.

**Methods:**

In the lung clinics of the Province of Yogyakarta, Indonesia, we recruited all consecutive patients with symptoms suggesting TB, aged ≥18 years. Routine TB examination consisted of clinical evaluation, sputum smear microscopy, and chest radiography. For research purposes, we added sputum culture, Human Immunodeficiency Virus (HIV) testing, and follow-up for 1.5 years or 2.5 years if culture results disagreed with the initial clinical diagnosis. The initial diagnosis was considered incorrect if patients did not respond to treatment. We calculated sensitivity and specificity of the TB routine examination using culture and a composite reference standard (CRS – a combination of routine examination, culture, and follow-up) as the reference standards. All analyses were conducted with IBM SPSS Statistics 25 (IBM Corp., Armonk, NY, USA).

**Results:**

Between 2013 and 2015, we included 360 participants, and 21 were excluded due to incomplete data. Among those analyzed, 115 were initially diagnosed with smear-positive TB, 12 with smear-negative TB, and 212 non-TB. In 15 study participants, the diagnosis was changed after median 45 (range: 14–870) days; 14 participants initially not diagnosed with TB were later diagnosed with TB, while one subject initially diagnosed with TB actually did not have TB. Compared with culture and CRS, TB routine examination had sensitivity of 85% (95%CI: 77–91) and 90% (95%CI: 84–94), and specificity of 86.3% (95%CI: 81–91) and 99.5% (95%CI: 97–100), respectively.

**Conclusions:**

A combination of clinical evaluation with sputum microscopy and chest radiography provided high sensitivity and specificity in diagnosing TB in lung clinics; in only 4.4% the diagnosis was incorrect. There is a need to improve routine TB diagnostic work by using clinical evaluation, sputum smear microscopy, and chest radiography all together in other settings, such as in primary health centers.

**Trial registration:**

NCT02219945, clinicaltrials.gov. Registered 19 August 2014 (retrospectively registered).

## Background

Indonesia is the country with the third largest TB burden in the world and has accounted for the second highest gap between notifications of TB cases and the best estimate of incident cases [1]. As in many low resource settings, TB is usually diagnosed by clinical examination, sputum smear microscopy, and chest radiography (CXR). Among the 442,172 new and relapsed pulmonary TB cases, only 54% were confirmed bacteriologically by microscopy or less often, by culture [1]. Culture examination is reserved for suspected drug resistance in patients failing to respond to treatment or in patients who relapse [2]. Other diagnostic tools such as Polymerase Chain Reaction (PCR) and Gene-Xpert are still difficult to access and without subsidy are unaffordable for the majority of patients.

The World Health Organization (WHO) criteria for pulmonary TB diagnosis include clinical symptoms and a CXR suggestive of TB, isolation of *Mycobacterium tuberculosis* (MTB) by culture or acid-fast bacilli by sputum smear microscopy if culture is unavailable [3]. Clinicians might need to make a tentative diagnosis if clinical symptoms and CXR suggest TB while microscopy remains negative, or if the patient does not respond to the treatment of an alternative pulmonary diagnosis [3]. Patients with a clinical response to TB treatment are likely to have suffered from TB, although occasionally clinical improvement may occur in patients with sarcoid, cryptogenic organizing pneumonia or non-tuberculous mycobacteria (NTM) infection.

Establishing a correct diagnosis is challenging [4]. Symptoms have low sensitivity and specificity; CXR of NTM lung disease and lung cancer may mimic TB, and bacteriology examinations sometimes fail [4–6]. Only a few studies have evaluated the sensitivity and specificity of TB diagnosis in the routine settings of low- and middle income countries [7, 8].

The government of Indonesia established the latest national strategy for TB control in 2016, emphasizing reinforcement of TB programs’ leadership, escalation of quality of TB services (prevention, diagnosis and treatment), management of risk factors for TB, enhancement of collaborations and community participation, and reinforcement of TB program management [9].

The sensitivity and specificity of a routine work-up for suspected TB in Indonesia, particularly in lung clinics, have not been studied. Meanwhile, this kind of study could provide information for the government about the quality of routine TB service and how to improve it, thus eventually it could support decision making and escalate the quality of the national TB program. We included culture examination and long-term follow-up in order to evaluate the sensitivity and specificity of the diagnostic algorithm under service conditions.

## Methods

### Study aim and design

We aimed to investigate the sensitivity and specificity of routine TB diagnostic work-up in lung clinics in Indonesia. This research was a cohort study of patients suspected to have TB in Yogyakarta, Indonesia.

### Study setting

Yogyakarta Province had a population of 3,679,176 in 2015 [10]. It had 5 public lung clinics that provide services for lung diseases, predominantly TB, and where more than half of suspected TB patients in Yogyakarta were screened [11]. In 2016, after our study was completed, the lung clinics were integrated into one lung hospital. Patients were either self-referred or referred by primary health centers. The lung clinics used microscopy, CXR, and HIV Voluntary Counseling and Testing services. The province has 21 primary health centers that mostly have sputum smear microscopy facilities; few among them have CXR facilities [12]. The total TB case notification rate in Yogyakarta in 2015 was 73/100,000 [10].

### Study population

This study was part of a research to investigate the diagnostic sensitivity and specificity of an electronic nose in Yogyakarta, Indonesia (eNose study-NCT02219945). The study population consisted of TB suspects aged 18 years and older, who agreed to participate in the eNose study. They were enrolled from October 2013 to December 2015.

### Study parameters

As part of routine examination, spot-morning-spot smear microscopy and CXR were conducted. For research purposes, we added sputum culture, HIV testing, and followed the study participants over time. Following the normal procedures, all study participants attended the Lung Clinics for two consecutive days to have their diagnosis established by the Lung Clinics’ physicians. On the first day, patients underwent clinical examination, CXR, microscopy examination from a spot sputum specimen, and HIV testing. On the second day, patients collected morning and spot sputum specimens for microscopy, and for research purpose, another morning sputum specimen for microscopy and culture in the Microbiology Laboratory, Faculty of Medicine, Public Health and Nursing, Universitas Gadjah Mada, Indonesia. As part of the ongoing research, all TB suspects were prospectively followed up at their home, lung clinics, or by phone for 1.5 years after diagnosis. Patients were followed up for 2.5 years when the culture results disagreed with the initial clinical diagnosis (i.e. culture was positive for MTB, but the clinical diagnosis was non-TB, or culture was negative but patient was diagnosed with TB).

We recorded information about previous TB treatment, demographics (age, sex, and Body Mass Index - BMI), housing conditions, bacteriological examination, CXR reading, follow up of clinical symptoms, and comorbidities (HIV or Type 2 Diabetes Mellitus/T2DM). T2DM diagnosis was based on the national guidelines [13]. A.M.S. and F.S. double-entered all data into a database, and ensured no missing data or typing errors.

Sputum microscopy and culture examinations with Löwenstein-Jensen(LJ) medium were conducted according to the WHO laboratory guidelines [14]. For research purposes, one independent physician (TsW - a pulmonologist) re-read the CXRs, which were scored as suggestive for TB, possible TB, abnormal but no TB, and normal. Patients who were lost to follow-up at any point in time, or who had incomplete results of any diagnostic test were excluded from the study. Results of TB routine examination were available to those seeing the patients during follow-up, but not to the laboratory personnel.

The initial diagnosis (TB or non-TB) was considered incorrect and was revised in the referral health centers when patients did not respond to treatment, or if an alternative diagnosis was made during follow-up. A composite reference standard (CRS) [15, 16] which consists of symptoms, sputum microscopy and culture, CXR, and follow-up determined the final diagnostic classification (TB or non-TB).

A TB case was defined as: [1] patient with bacteriological confirmation and clinical illness or CXR suggestive of TB, and responding to TB treatment; or [2] patients without bacteriological confirmation, but with clinical illness and CXR suggestive of TB, and responding to TB treatment. At the end of study period, the patients were then divided into two groups: [1] patients whose diagnoses were not revised, and [2] patients who had their definitive diagnosis changed compared to their initial diagnoses.

### Data analysis

Previous studies revealed that among all TB symptoms, cough, weight loss, and night sweats were independent predictors of TB with or without HIV coinfection, [17–19] thus we confined the analysis to these symptoms. The CXRs were dichotomized: CXR suggestive for TB or possible TB were scored as “suggestive for TB”, while a CXR abnormal but no TB, and CXR considered normal were scored as “not suggestive of TB”. We calculated the proportion of revised diagnoses, and the sensitivity, specificity, and positive and negative predictive values (PPV and NPV) of the TB routine examination using culture and the CRS as the reference tests. We also investigated which factors were associated with the revision of diagnosis from non-TB into TB by calculating the relative risks (RR) and their 95% confidence intervals (95%CI). The RR was considered significant if the 95%CI did not contain value of 1.00. All analyses were conducted with IBM SPSS Statistics 25 (IBM Corp., Armonk, NY, USA).

## Results

**Fig. 1:**
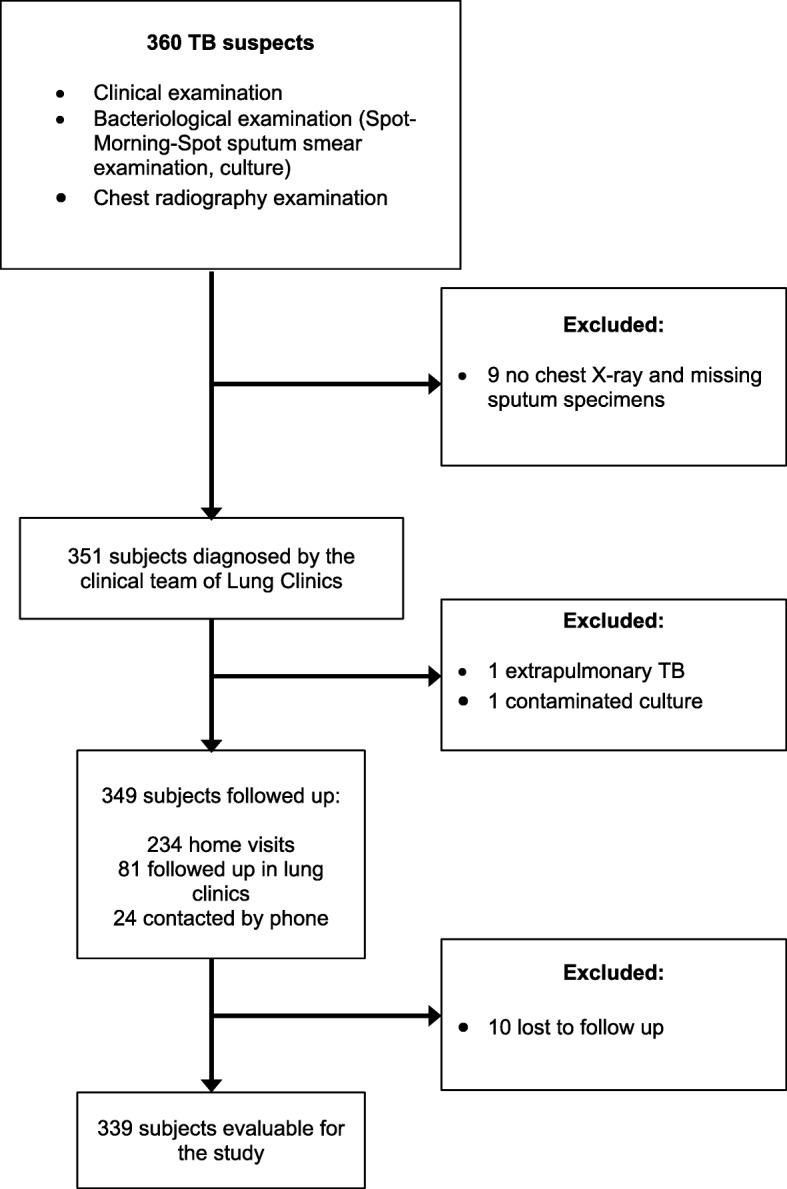
Flow chart of study participants

In all, 360 consecutive TB suspects were prospectively included. Twenty-one subjects were excluded for various reasons (Fig. 1). Most of the subjects were male, with median age 46 (range: 18–87) years, and normal BMI (18.5-25 kg/m^2^). Most subjects (79%) lived in a crowded neighborhood or poorly ventilated housing, 7% of them had TB index cases in their surrounding (house-hold members, colleagues, or close neighbors), 12% of them had a history of previous TB, 2.4% had HIV infection, 7.7% had T2DM, and 32% were current smokers.

Out of 339 participants, 115 were initially diagnosed with smear-positive TB, 12 had smear-negative TB, and 212 had diagnoses other than TB (asthma, pneumonia, bronchiectasis, chronic bronchitis, Chronic Obstructive Pulmonary Diseases, Obstructive Syndrome Post TB, lung fibrosis, lung abscess, lung cancer, pleural effusion, and polycystic lung disease). Of the eight patients who had HIV infection, 4 patients tested positive for TB in smear microscopy; 2 patients were diagnosed with TB but were smear negative and the other 2 patients were diagnosed as non-TB.

Table 1 shows that when culture and follow-up were used in parallel with TB routine examination to establish the final diagnosis, the final diagnosis changed for 15 (4.4%) study participants; 14 more study participants in retrospect had TB now, while only one subject initially diagnosed with TB actually did not have TB.

**Table 1:** Characteristics of study participants

Characteristics	Overall (*n* = 339)	Diagnosis not revised (*n* = 324)		Diagnosis revised (*n* = 15)	
		TB (*n* = 126)	Non-TB (*n* = 198)	Non-TB to TB (*n* = 14)	TB to Non-TB (*n* = 1)
Symptoms, n(%)					
Cough>2 weeks	329 (97.1)	122 (96.8)	192 (97)	14 (100)	1 (100)
Unintentional weight loss	211 (62.2)	109 (86.5)	92 (46.5)	9 (64.3)	1 (100)
Night sweats	167 (49.3)	92 (73.0)	69 (34.8)	5 (35.7)	1 (100)
Sputum smear microscopy, n(%)					
Positive	115 (33.9)	114 (90.5)	0 (0)	0 (0)	1 (100)
Negative	224 (66.1)	12 (9.5)	198 (100)	14 (100)	0 (0)
Mycobacterial culture, n(%)					
*M. tuberculosis*	113 (33.3)	96 (76.2)	7 (3.5)	10 (71.4)	0 (0)
NTM*	17 (5)	11 (8.7)	6 (3)	0 (0)	0 (0)
Negative	209 (61.7)	19 (15.1)	185 (93.4)	4 (28.6)	1 (100)
Chest X-ray findings at presentation, n(%)					
Suggestive of TB	158 (46.6)	117 (92.9)	33 (16.7)	7 (50)	1 (100)
Not suggestive of TB	181 (53.4)	9 (7.1)	165 (83.3)	7 (50)	0 (0)
Follow-up, n(%)					
Suggestive of TB	140 (41.3)	126 (100)	0 (0)	14 (100)	0 (0)
Not suggestive of TB	199 (58.7)	0 (0)	198 (100)	0 (0)	1 (100)

*NTM: Non-Tuberculous Mycobacteria

Fourteen patients were initially diagnosed as non-TB (7 were diagnosed with bacterial pneumonia, 3 with chronic bronchitis, 2 with post TB sequelae, 1 with bronchiectasis, and 1 with bronchopneumonia), but then their clinical conditions deteriorated and after median 83 (range: 14–870) days, 13 out of 14 patients were diagnosed as pulmonary TB, and 1 patient was diagnosed as Multi Drug Resistant Tuberculosis (MDR-TB). One of these pulmonary TB patients was HIV co-infected. One patient who was initially diagnosed with TB suffered from drug-induced liver toxicity at week 6 of treatment, and his TB drugs had to be stopped. He was referred to the hospital and obtained other non-TB drugs for one month, and afterwards, when sputum microscopy appeared negative, the treatment was stopped and he has remained well since; we therefore concluded that he probably did not have TB. Two other TB patients were considered cured, but then relapsed and obtained a re-treatment TB regimen.

Among non-TB patients whose diagnoses were revised into TB, six patients with CXR suggesting TB developed TB after around one month, and one patient with CXR suggesting TB developed TB after 26 months. Seven patients who had CXR not suggesting TB developed TB around 9 months after their initial diagnostic work-up.

Seven non-TB patients had MTB cultured in a sputum specimen, but they did not develop any TB symptoms during follow-up. Thus, we suspected them to have false-positive culture results. Three out of these 7 patients had a history of previous TB. Among 30 TB patients whose diagnoses were not revised, 19 patients had negative culture, and in 11 patients, sputum culture grew NTM. They responded to TB treatment and all of them were considered cured. Therefore, these patients were suspected to have false-negative cultures. In two patients, probably a clerical error occurred, with results exchanged between them. Samples of these two patients were processed in the same day; one had positive smear microscopy for TB, while the other had a negative microscopy test. The patient who was smear-positive and negative by culture was treated with anti TB drugs and subsequently recovered, whilst the other patient whose smear was negative and whose culture was positive did not get any TB treatment and did not deteriorate over time. Moreover, we noticed from the laboratory notes that three non-TB patients with negative culture whose diagnoses were revised into TB collected saliva instead of sputum, and sample specimens were too small (1 ml while > 2 ml is required). Most of patients who were suspected to have false-negative culture collected saliva instead of sputum, and with insufficient volume (< 2 ml).

The TB routine examinations in lung clinics in Yogyakarta had sensitivity of 85% (95%CI: 77–91) and 90% (95%CI: 84–94), and specificity of 86.3% (95%CI: 81–91) and 99.5% (95%CI: 97–100), taking culture and CRS as the reference tests, respectively (Table 2). Sensitivity and specificity of different symptoms and diagnostic tests were various, using CRS as the reference test (Table 3). Table 4 shows that patients with TB index cases in their surroundings were 12 times more likely to have revision of diagnosis from non-TB into TB compared to patients without TB index cases in their surroundings. Patients with a positive culture were 29 times more likely to have revision of diagnosis than patients with negative culture, and patients with CXR suggesting TB were 4 times more likely to have revision of diagnosis than patients with CXR not suggesting TB. Number of patients whose diagnoses were revised is too low to conduct a multivariate analysis.

**Table 2:** Performance of TB routine examination for TB diagnosis

	Sensitivity (95%CI)	Specificity (95%CI)	PPV (95%CI)	NPV (95%CI)
TB routine examination (culture as the reference test)	85 (77–91)	86.3 (81.1–90.5)	75.6 (68.9–81.3)	92 (88.1–94.7)
TB routine examination (composite reference standard as the reference test)	90 (83.8–94.4)	99.5 (97.2–99.9)	99.2 (94.7–99.9)	93.4 (89.6–95.9)

**Table 3:** Sensitivity and specificity of different diagnostic tools, using CRS as the reference test

Diagnostic tool	Sensitivity (95%CI)	Specificity (95%CI)
Symptoms		
Cough>2wks	97.1 (92.9–99.2)	10.6 (6.7–15.7)
Unintentional weight loss	84.3 (77.2–89.9)	53.3 (46.1–60.4)
Night sweats	69.3 (60.9–76.8)	64.8 (57.8–71.4)
Cough>2wks + weight loss	82.1 (74.8–88.1)	57.3 (50.1–64.3)
Cough> 2wks + night sweats	67.1 (58.7–74.8)	67.3 (60.4–73.8)
Weight loss + night sweats	65.0 (56.5–72.9)	76.9 (70.4–82.6)
All symptoms	62.9 (54.3–70.9)	78.4 (72.0–83.9)
Sputum Smear Microscopy (with Spot-Morning-Spot specimens)		
Positive for AFB	81.4 (74–87.5)	99.5 (97.2–99.9)
Chest radiography (CXR)		
Abnormalities suggestive of active TB	88.6 (82.1–93.3)	82.9 (77–87.9)
Combination		
Cough>2wks + SSM	80.0 (72.4–86.3)	99.5 (97.2–99.9)
Cough>2wks + CXR	86.4 (79.6–91.6)	85.4 (79.8–90.0)
Weight loss+SSM	71.4 (63.2–78.7)	99.5 (97.2–99.9)
Weight loss+CXR	76.4 (68.5–83.2)	91.0 (86.1–94.6)
Night sweat+SSM	60.0 (51.4–68.2)	99.5 (97.2–99.9)
Night sweat+CXR	63.6 (55–71.5)	95.5 (91.6–97.9)
Cough>2wks + weight loss+SSM	70.0 (61.7–77.5)	99.5 (97.2–99.9)
Cough>2wks + weight loss+CXR	74.3 (66.2–81.3)	92.5 (87.9–95.7)
Cough>2wks + night sweat+SSM	58.6 (50–66.8)	99.5 (97.2–99.9)
Cough> 2wks + night sweat+CXR	61.4 (52.8–69.5)	96.0 (92.2–98.3)
Weight loss+night sweat+SSM	56.4 (47.8–64.8)	99.5 (97.2–99.9)
Weight loss+night sweat+CXR	59.3 (50.7–67.5)	96.5 (92.9–98.6)
All symptoms+SSM	55 (46.4–63.4)	99.5 (97.2–99.9)
All symptoms+CXR	57.1 (48.5–65.5)	97.0 (93.6–98.9)
SSM + CXR	78.6 (70.8–85.1)	99.5 (97.2–99.9)
Cough>2wks + SSM + CXR	77.1 (69.3–83.8)	99.5 (97.2–99.9)
Weight loss+SSM + CXR	68.6 (60.2–76.2)	99.5 (97.2–99.9)
Night sweat+SSM + CXR	58.6 (50–66.8)	99.5 (97.2–99.9)
Cough>2wks + weight loss+SSM + CXR	67.1 (58.7–74.8)	99.5 (97.2–99.9)
Cough>2wks + night sweat+SSM + CXR	57.1 (48.5–65.5)	99.5 (97.2–99.9)
Weight loss+night sweat+SSM + CXR	55.0 (46.4–63.4)	99.5 (97.2–99.9)
All symptoms+SSM + CXR	53.6 (45–62)	99.5 (97.2–99.9)

**Table 4:** Factors associated with the revision of diagnosis from non-TB into TB among non-TB patients

Variable	Revised diagnosis from non-TB into TB		RR	95%CI
	Yes (*n* = 14)	No (*n* = 198)		
Age, n (%)				
≥46 years old	5 (35.7)	117 (59.1)	0.41	0.14–1.18
< 46 years old	9 (64.3)	81 (40.9)	1	
Sex, n (%)				
Male	8 (57.1)	118 (59.6)	0.91	0.33–2.53
Female	6 (42.9)	80 (40.4)	1	
BMI, n (%)				
< 18.5 or > 25	6 (42.9)	75 (37.9)	1.21	0.44–3.37
18.5–25	8 (57.1)	123 (62.1)	1	
History of TB, n (%)				
Yes	2 (14.3)	26 (13.1)	1.10	0.26–4.64
No	12 (85.7)	172 (86.9)	1	
Housing conditions, n (%)				
Crowded neighborhood or poorly ventilated house	12 (85.7)	136 (68.7)	2.59	0.60–11.26
Not crowded neighborhood and adequate ventilated house	2 (14.3)	62 (31.3)	1	
TB index cases in surrounding area, n(%)				
Present	7 (50)	9 (4.5)	12.25	4.90–30.60
Not present	7 (50)	189 (95.5)	1	
HIV* comorbidities, n(%)				
Yes	1 (7.1)	2 (1.0)	5.36	0.99–28.89
No	13 (92.9)	196 (99.0)	1	
Diabetes mellitus comorbidities, n(%)				
Yes	1 (7.1)	9 (4.5)	1.55	0.23–10.73
No	13 (92.9)	189 (95.5)	1	
Smoking status, n(%)				
Current smoker or stopped smoking < 10 years	3 (21.4)	55 (27.8)	0.72	0.21–2.50
Never-smoker or non-smoker > 10 years	11 (78.6)	143 (72.2)	1	
Symptoms, n(%)				
*Cough > 2 weeks*				
Yes	14 (100.0)	177 (89.4)	N.A.	N.A.
No	0 (0.0)	21 (10.6)		
*Unintentional weight loss*				
Yes	9 (64.3)	92 (46.5)	1.98	0.69–5.71
No	5 (35.7)	106 (53.5)	1	
*Night sweats*				
Yes	5 (35.7)	162 (81.8)	0.57	0.20–1.67
No	9 (64.3)	36 (18.2)	1	
Sputum smear microscopy, n(%)				
Positive	0 (0.0)	0 (0.0)	N.A.	N.A.
Negative	14 (100.0)	198 (100.0)		
Mycobacterial culture, n(%)				
*M. tuberculosis*	10 (71.4)	7 (3.5)	28.68	10.05–81.81
Negative or NTM*	4 (28.6)	191 (96.5)	1	
Chest X-ray findings at presentation, n(%)				
Suggestive of TB	7 (50.0)	33 (16.7)	4.30	1.60–11.57
Not suggestive of TB	7 (50.0)	165 (83.3)	1	
Follow-up, n(%)				
Suggestive of TB	14 (100.0)	0 (0.0)	N.A.	N.A.
Not suggestive of TB	0 (0.0)	198 (100.0)		

*HIV: Human Immunodeficiency Virus, NTM: Non-Tuberculous Mycobacteria

Data are presented as percentage

## Discussion

TB routine examinations in Yogyakarta lung clinics had high sensitivity and specificity. Only 4.1% of 339 consecutively enrolled study participants who were initially not diagnosed with TB later turned out to have TB. To our knowledge this study is the first report addressing sensitivity and specificity of TB diagnosis under routine conditions in lung clinics in Indonesia. Follow-up as a part of a composite reference standard to assess diagnostic test characteristics has been successfully used in many different settings [16, 17, 20, 21].

The diagnostic sensitivity and specificity in this study are higher compared to the study from Kwazulu-Natal, South Africa [8]. All 12 patients in our study who were bacteriologically negative but clinically diagnosed with TB, had clinical improvement during the TB treatment. Boehme et al. found that only 67 out of 138 patients who were clinically diagnosed with TB despite negative bacteriology improved after TB treatment [7]. Using culture as reference test, a study in smear negative TB patients in Pakistan showed that the clinical diagnosis was correct in 80% [22]. In a retrospective study in Taiwan, 87% of smear negative patients who were clinically diagnosed with TB were confirmed to have TB based on their response to therapy [6]. Some factors may explain this: Indonesia has higher TB prevalence which results in higher pretest probability; the lung clinics conducted multiple tests in the first examination, thus the physicians had more data to support correct diagnosis; and Indonesia has lower HIV prevalence [1], which contributes to the higher sensitivity of sputum microscopy and CXR.

In Yogyakarta, Indonesia, the lung clinics had the facilities needed to establish a TB diagnosis, meanwhile some of the primary health centers did not have laboratories facilities for sputum smear microscopy or CXR, thus they needed to send samples or patients for microscopy or CXR examinations to the lung clinics or higher-level hospitals. In addition, the lung clinics conducted both microscopy and CXR examinations in the first visit, while primary health centers followed the national diagnostic algorithm that recommends CXR examination only when sputum smears are negative. Therefore, the duration of diagnostic delay for TB diagnosis in primary health centers was significantly longer than in lung clinics [23], and the primary health centers may not have sufficient tools to establish the correct diagnosis. However, there have been no studies that investigated the sensitivity and specificity of TB diagnosis in the primary health centers in Indonesia, thus we could not compare the results with the lung clinics. Furthermore, the proportion of patients lost in primary health centers was higher than in lung clinics, due to this longer duration of the diagnostic process [23], although generally the distances between patients’ houses and primary health centers were closer than distances between their houses and lung clinics. Often patients needed to come more frequently to the primary health centers than to lung clinics to get diagnosed, which means more costs and time spent, thus causing patients’ reluctance to finish the diagnostic procedures [23]. Indeed, there is a high gap (estimated around 47%) between notification and the estimation of incident TB cases due to undetected and underreported cases in Indonesia, including Yogyakarta [1]. Therefore, if the TB diagnostic process in the lung clinics could be replicated in the primary health centers, it would improve the detection rate and reduce further TB transmission in the community.

Presence of all symptoms showed fairly good sensitivity and specificity as reported earlier from Tanzania [18], and it helped to establish an accurate diagnosis. Persistent cough of at least 2 weeks duration had the highest sensitivity, although other studies have reported low sensitivity and high specificity for persistent cough [17, 24]. Night sweats were associated with the highest specificity. Indeed, night sweats have generally been considered as a specific symptom for TB though the pathophysiology is poorly understood; [25, 26] night sweats are limited to the upper trunk in TB [27].

It is reasonable that a revision of diagnosis from non-TB into TB was associated with a presence of TB index cases in patient’s surroundings, as the presence of exposure to TB index cases is one of risk factors to contract TB [1, 9]. Culture examination is indeed considered the gold standard for the diagnosis of TB; 10 out of 14 patients whose diagnoses were revised had positive culture but negative smear. Nonetheless, the solid media used for culture in our study typically has a slow turn-around time, considerably longer than cultures in liquid media that have the additional advantage of improved sensitivity. Liquid culture may however have lower specificity due to higher contamination rates [28–30]. To increase sensitivity of LJ culture, 2–3 specimens per patient should preferably be examined to improve the diagnostic yield [31, 32]. In only 34 participants in our study, multiple specimens were submitted for culture. CXR has higher specificity and sensitivity compared to clinical symptoms, which corresponds with previous studies [24, 33]. CXR in TB may resemble other pulmonary conditions [4, 5], however, almost all non-TB patients whose diagnoses were revised into TB and who had CXR suggesting TB, developed TB after a short time. Our data illustrate that CXR is a helpful tool to screen for active TB. A previous study reported that ≥80% of patients with pulmonary TB have at least one among five different radiographic appearances [19].

According to the TB national guidelines in Indonesia, patients with negative smear should return or be followed-up to investigate their responses to the antibiotics given [2]. However, in reality, patients often do not return to the health centers, and the patients who are lost are not tracked [23]. Many negative-sputum-smeared patients in our study did not visit the lung clinics or other health centers after their initial diagnosis. In this situation, we note that follow-up helped to establish a correct TB diagnosis. A national study in 2017 revealed that around 20% of new cases were not diagnosed [1]. Follow-up also provides an affordable way to evaluate the sensitivity and specificity of the diagnostic process in low resource settings.

This study is the first report on the proportion of false positive cultures in Indonesia. We suspect that the rate of false (positive and negative) culture results was 11.2%, while our estimate of false positive culture rate is 2%, which is comparable with earlier reports in high and middle/low-income countries [34–36]. To confirm suspected cross-contamination resulting in false-positive diagnoses, fingerprinting techniques help to identify similarity of strains from patients that have no epidemiological link. For resource-poor settings like Indonesia, these techniques are currently unaffordable but clinical laboratories can improve operational procedures by enhancing adherence to protocols [31], and participating in External Quality Assessment programs. Despite unavailability of fingerprinting analysis, we assessed the possibility of false-positive culture in our study through all available data. The lack of quality and quantity of samples might influence the quality of culture in these suspected false culture cases. Therefore, laboratory and other health care workers should instruct patients how to produce sufficient samples to increase the sample quality and detection rate [37]. In a similar setting in Java, Indonesia, education provided for sputum collection for the patients was suboptimal [38].

To address the diagnostic delay, a rapid, reliable, and point-of-care diagnostic tool is needed. However, clinicians need to be conscious of the possibility of false-positive test results of any diagnostic tool, and clinical judgment remains important. Some tools are now under development, such as the loop-mediated isothermal amplification test for TB [1], and electronic nose [39]. In the settings of lung clinics and primary health centers in Yogyakarta and elsewhere in Indonesia, Gene-Xpert TB/RIF is not readily available; it is available in some hospitals [40], and although the use of Gene-Xpert could increase the TB positivity rate [40], the relatively high cost of cartridges precludes extensive use if suspicion for drug resistance is low. False-positive test results for TB may also occur with PCR [41] or Gene-Xpert [42].

While waiting for an implementation of a rapid, reliable, and point-of-care TB diagnostic tool, optimizing the current routine TB diagnostic work is a reasonable option. The current national diagnostic algorithm that recommends CXR examination when sputum smear results are negative, was proven to delay the TB diagnosis, and one of the factors causing patient’s loss, although it was originally developed to improve the sensitivity and specificity of TB diagnosis [23]. Our study showed that the diagnostic process in the lung clinics which employs clinical evaluation, sputum smear microscopy, and chest radiography all together had high sensitivity and specificity, and in the same time reduced the delay time of TB diagnosis, as indicated by a previous study [23]. Therefore, an attempt should be done to replicate the diagnostic process in the lung clinics to other settings, such as primary health centers. If laboratory facilities could not be provided in the primary health centers, there should be a prompt referral system that enables patients to get microscopy and CXR examinations on the same day. This effort will reduce the rate of patient loss thus reducing the number of undetected cases and ongoing TB transmission.

There are some limitations in this study; in particular, it was performed in Yogyakarta province alone. The organization of lung clinics in Yogyakarta is however typical and representative for lung clinics in Indonesia and we therefore assume that the diagnostic sensitivity and specificity of TB routine examination is comparable to other lung clinics in Indonesia. Another limitation was that we used solid (LJ) culture medium, which has lower sensitivity compared to liquid (e.g. Mycobacteria Growth Indicator Tube) culture, and we used only one specimen for culture. We did not perform fingerprinting so that we could not further confirm the suspected false positive culture results, and we did not perform Drug Susceptibility Testing (DST) on every specimen; however, we did not detect study participants failing to respond to TB treatment and the MDR-TB prevalence in Indonesia is low [1], thus for that matter, the lack of DST did not confound our assessment.

## Conclusion

In summary, a combination of clinical evaluation with sputum microscopy and chest radiography in lung clinics provided high sensitivity and specificity in diagnosing TB; in only 4.4%, the diagnosis was incorrect. While waiting for an implementation of point-of-care, fast, accurate, and easy-to-use TB diagnostic tool, there is a need to improve routine TB diagnostic work by using clinical evaluation, sputum smear microscopy, and chest radiography all together in other settings, such as in primary health centers.

## Abbreviations

95%CI: 95% Confidence Interval
BMI: Body Mass Index
CRS: Composite Reference Standard
CXR: Chest radiography
DST: Drug Susceptibility Testing
HIV: Human Immunodeficiency Virus
LJ: Löwenstein-Jensen
MDR-TB: Multi Drug Resistant Tuberculosis
MTB: *Mycobacterium tuberculosis*
NPV: negative predictive values
NTM: non-tuberculous mycobacteria
PBS: Phosphate-buffered saline
PCR: Polymerase Chain Reaction
PPV: positive predictive values
RR: Relative Risk
T2DM: Type 2 Diabetes Mellitus
TB: Tuberculosis
WHO: World Health Organization
